# Revised Adult T-Cell Leukemia-Lymphoma International Consensus Meeting Report

**DOI:** 10.1200/JCO.18.00501

**Published:** 2019-01-18

**Authors:** Lucy B. Cook, Shigeo Fuji, Olivier Hermine, Ali Bazarbachi, Juan Carlos Ramos, Lee Ratner, Steve Horwitz, Paul Fields, Alina Tanase, Horia Bumbea, Kate Cwynarski, Graham Taylor, Thomas A. Waldmann, Achilea Bittencourt, Ambroise Marcais, Felipe Suarez, David Sibon, Adrienne Phillips, Matthew Lunning, Reza Farid, Yoshitaka Imaizumi, Ilseung Choi, Takashi Ishida, Kenji Ishitsuka, Takuya Fukushima, Kaoru Uchimaru, Akifumi Takaori-Kondo, Yoshiki Tokura, Atae Utsunomiya, Masao Matsuoka, Kunihiro Tsukasaki, Toshiki Watanabe

**Affiliations:** ^1^ Imperial College Healthcare National Health Service (NHS) Trust, London, United Kingdom; ^2^ Imperial College London, London, United Kingdom; ^3^ Osaka International Cancer Institute, Osaka, Japan; ^4^ Necker University Hospital, Paris, France; ^5^ American University of Beirut, Beirut, Lebanon; ^6^ University of Miami, Miami, FL; ^7^ Washington University School of Medicine, St Louis, MO; ^8^ Memorial Sloan Kettering Cancer Center, New York, NY; ^9^ Guys and St Thomas Hospital, Kings Health Partners, London, United Kingdom; ^10^ Fundeni Clinical Institute, Bucharest, Romania; ^11^ Emergency University Hospital, Bucharest, Romania; ^12^ University College London Hospitals NHS Trust, London, United Kingdom; ^13^ National Cancer Institute, Bethesda, MD; ^14^ Universidade Federal da Bahia, Salvador, Brazil; ^15^ Weill Cornell Medical College, New York, NY; ^16^ University of Nebraska, Omaha, NE; ^17^ Mashhad University of Medical Sciences, Mashhad, Iran; ^18^ Nagasaki University, Nagasaki, Japan; ^19^ National Kyushu Cancer Center, Fukuoka, Japan; ^20^ Iwate Medical University, Morioka, Japan; ^21^ Kagoshima University Hospital, Kagoshima, Japan; ^22^ University of the Ryukyus, Okinawa, Japan; ^23^ The University of Tokyo, Tokyo, Japan; ^24^ Kyoto University, Kyoto, Japan; ^25^ Hamamatsu University School of Medicine, Hamamatsu, Japan; ^26^ Imamura General Hospital, Kagoshima, Japan; ^27^ Kumamoto University, Kumamoto, Japan; ^28^ Saitama Medical University, Saitama, Japan

## Abstract

**Purpose:**

Adult T-cell leukemia-lymphoma (ATL) is a distinct mature T-cell malignancy caused by chronic infection with human T-lymphotropic virus type 1 with diverse clinical features and prognosis. ATL remains a challenging disease as a result of its diverse clinical features, multidrug resistance of malignant cells, frequent large tumor burden, hypercalcemia, and/or frequent opportunistic infection. In 2009, we published a consensus report to define prognostic factors, clinical subclassifications, treatment strategies, and response criteria. The 2009 consensus report has become the standard reference for clinical trials in ATL and a guide for clinical management. Since the last consensus there has been progress in the understanding of the molecular pathophysiology of ATL and risk-adapted treatment approaches.

**Methods:**

Reflecting these advances, ATL researchers and clinicians joined together at the 18th International Conference on Human Retrovirology—Human T-Lymphotropic Virus and Related Retroviruses—in Tokyo, Japan, March, 2017, to review evidence for current clinical practice and to update the consensus with a new focus on the subtype classification of cutaneous ATL, CNS lesions in aggressive ATL, management of elderly or transplantation-ineligible patients, and treatment strategies that incorporate up-front allogeneic hematopoietic stem-cell transplantation and novel agents.

**Results:**

As a result of lower-quality clinical evidence, a best practice approach was adopted and consensus statements agreed on by coauthors (> 90% agreement).

**Conclusion:**

This expert consensus highlights the need for additional clinical trials to develop novel standard therapies for the treatment of ATL

## INTRODUCTION

Adult T-cell leukemia-lymphoma (ATL) is an intractable mature T-cell malignancy with diverse clinical features, etiologically associated with a retrovirus designated human T-cell leukemia virus type I or human T-lymphotropic virus type 1 (HTLV-1), which is endemic in several regions, including the southwest region of Japan, Central and South America, central Africa, the Middle East, Far East, central Australia, and Romania.^1,2^ Because of population migration, sporadic cases are observed in North America, particularly in New York, NY, and Miami, FL; and Europe, mostly in France and the United Kingdom. Incidence of ATL is rising in nonendemic regions of the world.^3^ In 2009, ATL researchers joined together and published an ATL consensus report that has been a standard reference for clinical trials of new agents for ATL and that focused on definition, prognostic factors, clinical subtype classification, treatment, and response criteria.^4^

Since publication, additional progress has been made in the molecular pathophysiology of ATL and risk-adapted treatment approaches.^5^ The ATL clinical workshop held during the 18th International Conference on Human Retrovirology—HTLV and Related Viruses—held in Tokyo, Japan, March, 2017 focused on discussion and revision of the 2009 consensus report. Consensus methodology and its limitations are detailed in the Data Supplement.

Some therapeutic agents used in the treatment of ATL are not universally available and treatment strategies will therefore differ among countries, which is reflected in these recommendations (Table 1). For example, mogamulizumab and certain components of the vincristine, cyclophosphamide, doxorubicin, and prednisone (VCAP); doxorubicin, ranimustine, and prednisone (AMP); and vindesine, etoposide, carboplatin, and prednisone (VECP) chemotherapy regimen (modified LSG15) are presently unavailable outside of Japan, whereas zidovudine and interferon-alpha are not approved in Japan but can be used in other parts of the world. There is also variability in the availability of positron emission tomography/computed tomography (PET/CT) and various molecular diagnostic tools, although their usefulness remains mostly unproven. Whereas there is general consensus among experts that the treatments listed are appropriate (> 90% consensus), the level of evidence should be regarded as low or very low unless specifically listed—the equivalent of a GRADE evidence score of C or D, or National Comprehensive Cancer Network (NCCN) 2B—and the treatment recommendations (Table 1) reflect the best practice consensus of expert opinion. The current consensus report is not a guideline as in case of the 2009 consensus.^4^ An aim of this report is to recommend good practice where there is a limited evidence base but for which a degree of consensus or uniformity is likely to benefit patient care and may be used as a tool to assist policymakers.

**TABLE 1.**
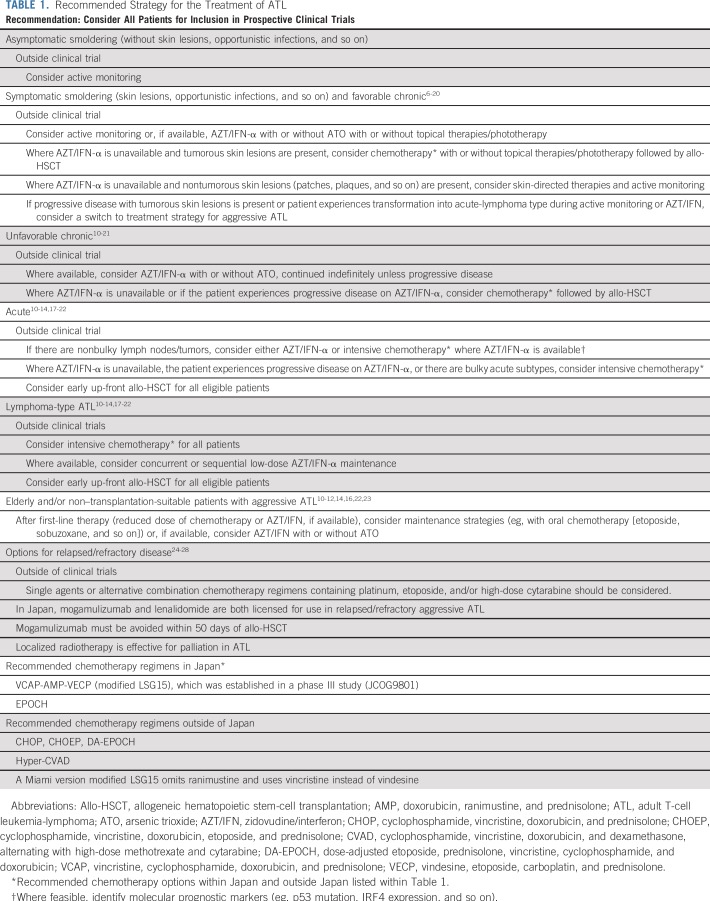
Recommended Strategy for the Treatment of ATL

## LYMPHOMA TYPE OF ATL, EXTRANODAL PRIMARY CUTANEOUS VARIANT

**FIG 1.**
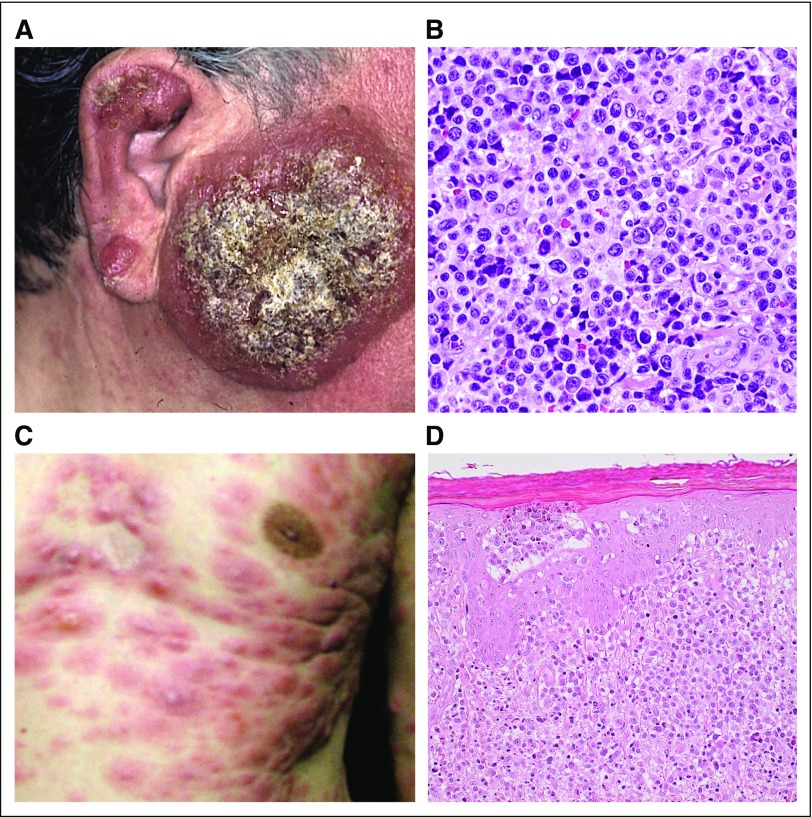
Primary cutaneous tumoral type (PCT) ATL. (A) Facial PCT with (B) histopathology that shows massive infiltration of pleomorphic lymphocytes in the dermis and subcutaneous tissue. (C) Nodulotumors of the chest with (D) histopathology that shows massive infiltration of atypical lymphocytes in the dermis and with epidermotropism.

Cutaneous lesions of ATL are variable and may resemble those of mycosis fungoides (MF), with mostly an indolent course, but some are associated with a poor prognosis. Therefore, ATL should be distinguished from cutaneous T-cell lymphomas, including MF, and peripheral T-cell lymphoma (PTCL), especially in endemic areas, by HTLV-1 serology and genomic analysis as necessary. In a large Japanese retrospective study of ATL with cutaneous lesions, 5-year survival rate was 0% in nodulotumoral and erythrodermic types compared with more than 40% in multipapular, plaque, and patch types.^6^ In the 2009 report, primary cutaneous tumoral (PCT) ATL without leukemic, lymph node, and other lesions was frequently included within smoldering ATL and was considered a poor prognostic factor by univariable analyses.^4,7^ PCT-ATL is distinct, with cutaneous lesions appearing as tumors that grow rapidly and whose histology shows large, atypical cells with a high proliferative index^7^ (Fig 1). In this revision, we agreed that watchful waiting is inappropriate in PCT-ATL as it frequently has a progressive and fatal clinical course that resembles aggressive ATL.^4,7^ Recently, Japanese hematologists, dermatologists, and pathologists proposed the entity lymphoma type of ATL, extranodal primary cutaneous variant, which shows a poor prognosis and includes PCT-ATL. Macroscopic findings are mostly nodulotumoral and pathologic findings show high-grade T-cell lymphoma type (pleomorphic, medium, or large size cells) with prominent perivascular infiltration and scant epidermotropism.^8^ Such cases could be considered for immediate treatment per aggressive ATL protocols with intensive chemotherapy with or without skin-directed therapies, including phototherapy or radiation followed by allogeneic hematopoietic stem-cell transplantation (allo-HSCT; see Allo-HSCT for Agressive ATL section) or interferon with zidovudine (IFN/AZT) with or without arsenic trioxide with or without skin-directed therapies. It should be noted, however, that precisely defining cutaneous tumoral type is difficult—size, height, number, with or without ulceration or subcutaneous extension, and so on—although the nodulotumoral type was defined as nodules or tumors with diameters > 1 cm and the multipapular type as multiple papules with a diameter < 1 cm.^6^ Furthermore, other types of cutaneous lesions can be aggressive.^6^ Papules, nodules, and tumors are considered as solid, palpable, and raised lesions with a diameter of < 1 cm, < 3 cm, and ≥ 3 , respectively. Papules usually occur as multiple lesions, whereas tumor(s) may be seen even as a solitary lesion. Recently, a prognostic index (PI) for chronic- and smoldering-type ATL identified soluble interleukin-2 receptor (sIL-2R) levels as an independent prognostic factor. sIL-2R may therefore be useful but requires additional prospective validation.^9^ Additional clinicopathologic and molecular studies are warranted in cutaneous ATL, including the application of recent new agents for MF and PTCL.^8^

### Consensus Statements

1. HTLV-1 serology should be undertaken in cases of cutaneous T-cell lymphoma and PTCL, particularly in HTLV-1 endemic regions.
2. Active monitoring is not appropriate for PCT-ATL, and intensive treatment should be considered.

## CNS LESIONS OF AGGRESSIVE ATL

Ten percent to twenty percent of patients with aggressive ATL will experience CNS progression.^29^ Thus, even in patients without a CNS lesion, it is important to incorporate CNS prophylaxis. Intrathecal prophylaxis was incorporated into sequential Japan Clinical Oncology Group (JCOG) studies for aggressive ATL (chemotherapy trials JCOG9303 and JCOG9801) following both the poor results of earlier study JCOG9109, which did not contain intrathecal prophylaxis,^10-13^ and a previous retrospective analysis that revealed that more than one half of relapses that occurred at a new site after chemotherapy occurred in the CNS.^30^ CNS involvement occurred in 1.6% of patients in JCOG9109 without intrathecal prophylaxis, in 6.3% of patients in JCOG9303 with intrathecal methotrexate (MTX)/prednisone, and in 3.5% of patients in the VCAP-AMP-VECP arm and 8.2% in the CHOP-14 arm (cyclophosphamide, doxorubicin, vincristine, prednisolone) of JCOG9801, both with intrathecal ara-C/MTX/prednisone(JCOG Prognostic Index and Characterization of Long-Term Survivors of Aggressive Adult T-Cell Leukaemia-Lymphoma [JCOG0902A]).^10-13^ The reason for the higher incidence in the JCOG9303 trial compared with the JCOG9109 trial might be associated with simultaneous CSF examination in all patients even without symptoms associated with CNS disease after one cycle of chemotherapy.^10,11^ In asymptomatic patients with aggressive ATL, diagnostic lumbar puncture/intrathecal chemotherapy should be performed at the end of the first chemotherapy or equivalent antiviral therapy (AZT/IFN) cycle upon successful disease control.

Options for treating active CNS disease at initial presentation include incorporating high-dose (HD) MTX into combination chemotherapy regimens (eg, CHOP-M or HD-MTX/HD cytarabine) for intracerebral tumors or adding intrathecal chemotherapy to the standard induction chemotherapy, as reported for other aggressive lymphomas.^31,32^ However, the scantiness of reports for these options for ATL precludes a more specific recommendation.

Retrospective analysis revealed that allo-HSCT with local treatment of ATL with CNS involvement achieved long progression-free survival (PFS) in four of 10 patients (> 2.5 years) despite high transplantation-related mortality (TRM).^7,33^ Numbers of such cases are small and the indication of allo-HSCT for ATL with CNS involvement remains controversial. No specific transplantation conditioning regimen can be recommended, but those that incorporate drugs that can cross the blood-brain barrier (eg, thiotepa) should be considered.

### Consensus Statement

1. Prophylactic CNS therapy should be considered for all patients with aggressive ATL.

## ALLO-HSCT FOR AGGRESSIVE ATL

The prognosis of aggressive ATL remains dismal with nontransplantation treatments.^1,6-16,21-26,29,34-38^ Nontransplantation treatment regimens alone are suboptimal for the majority of patients, although a proportion of patients with aggressive ATL who achieve complete remission after intensive chemotherapy or AZT/IFN therapy may achieve a long (> 5 years) PFS.^13,14^ After several promising case series were published,^17-19^ several large retrospective analyses of allo-HSCT in Japan reported favorable outcomes, with long-term survival achieved in approximately one third of allo-HSCT recipients after chemotherapy,^20,39^ but with significant TRM. The European Society for Blood and Bone Marrow Transplantation registry also demonstrated similar outcomes.^40^ Thus, treatment strategies that include allo-HSCT as consolidation are recommended in transplantation-eligible patients with aggressive ATL, although there have yet to be any prospective randomized trials to support this approach. Three PIs for aggressive ATL after chemotherapy have been reported as useful with validation cohorts: JCOG-PI, on the basis of corrected calcium and performance status (PS)^13^; ATL-PI, on the basis of age, serum albumin, sIL-2R,^41^ Ann Arbor stage, and PS; and modified ATL-PI, on the basis of corrected calcium, clinical subtype, PS, C-reactive protein, and sIL-2R level.^39^ However, long-term overall survival (OS) in the low-risk group was still poor and a group for whom up-front allo-HSCT might not be recommended could not be clearly identified. A proportion of patients with ATL with localized lymphoma had a long PFS after chemotherapy as well as chronic and a subgroup of acute cases treated with IFN/AZT.^13,14,41^ Several reports demonstrated a graft-versus-ATL effect which contributed to long-term relapse-free survival.^19,42,43^ ATL with abnormalities in tumor suppressor genes, such as p53, was reportedly resistant to IFN/AZT therapy and chemotherapy.^44,45^

As responses to intensive chemotherapy in general are not durable and long-term continuation of intensive chemotherapy is not feasible because of complications and cumulative toxicities, early allo-HSCT is recommended after response to first-line therapy.^46^ Thus, early referral to a transplantation center at diagnosis is strongly recommended, particularly in patients with high-risk features as described.^11,39,41^ With progressive disease, clinical outcome after allo-HSCT is poor^47,48^ and it is crucial to conduct an up-front allo-HSCT while ATL is controlled to maximize the cure rate. It is recommended that allo-HSCT for progressive disease be performed within the setting of a prospective clinical trial to investigate novel HSCT conditioning regimens or post-HSCT treatment strategies to improve current limited treatment results. When considering allo-HSCT, the standard approach involves searching for an HLA-matched related donor (MRD) or an HLA-matched unrelated donor (MUD) at diagnosis. However, only a proportion of patients will have an MRD. HTLV-1 seronegative donors are also preferred to avoid the risk of donor-derived ATL.^49^ When only HTLV-1 seropositive related donors are available, it is recommended to exclude the presence of abnormally abundant HTLV-1–infected clones using Southern blotting or polymerase chain reaction on the basis of clonality methods.^50,51^ Furthermore, outside of Japan, HTLV-1 infection often arises in minority immigrant populations, which makes it difficult to obtain a suitable MUD from registry panels.^52,53^ In these cases, other possible approaches include cord blood transplantation (CBT) or haploidentical HSCT (haplo-HSCT). Early experience with haplo-HSCT or CBT was unsatisfactory; however, protocols for both CBT and haplo-HSCT have been modified in recent years, although the efficacy of these protocols in ATL is not known. Preliminary anecdotal experience suggests that the TRM remains high, although several reports have suggested that CBT provides results that are similar to other transplantation procedures for selected patients with ATL.^54-56^ Although experimental in the ATL setting, the addition of post-transplantation cyclophosphamide to haplo-HSCT protocols has improved transplantation outcomes for other hematologic malignancies. HSCT from alternative donor sources earlier in the disease course with good disease control may increase the potential to achieve outcomes that are similar to MUD transplantation outcomes. Without additional evidence from the results of ongoing trials (Data Supplement), it remains difficult to prioritize one donor source over another when an MRD or MUD is unavailable.

Both myeloablative and reduced-intensity conditioning (RIC) have been used in patients with ATL; however, as the median age in Japan is > 60 years, RIC regimens are increasingly used and sequential prospective trials have revealed the relative safety and promising efficacy of allo-HSCT with RIC.^57-59^ Intensity of conditioning should be determined by attendant comorbidities and patient fitness at transplantation.

Although allo-HSCT has the potential to cure ATL, relapse/progression of ATL after allo-HSCT remains a major obstacle and conveys a poor prognosis. In patients with focal relapse (eg, solitary lymph node or skin) radiotherapy alone with or without the reduction of immune suppression or donor lymphocyte infusions can achieve durable disease control.^60^ The roles of AZT/IFN in this setting or in prophylaxis to prevent relapse are yet to be determined.

In Japan, where mogamulizumab and lenalidomide are available for the treatment of relapsed/progressed ATL, reports after allo-HSCT remain limited. In patients with aggressive ATL who received mogamulizumab before allo-HSCT, there seems to be a significantly increased risk of severe and steroid-refractory graft-versus-host disease (GVHD).^48,61^ In general, if up-front allo-HSCT is planned after induction chemotherapy, intensive chemotherapy without mogamulizumab should be considered as a result of the described risk of GVHD. For relapsed/refractory patients treated with mogamulizumab, the interval between the last salvage mogamulizumab and allo-HSCT should be long (at least 50 days) to decrease drug levels in vivo^48,61^ and additional intensification of GVHD prophylaxis should be considered. Data on allo-HSCT after up-front IFN/AZT are limited, but allo-HSCT is generally feasible and recommended for patients with acute ATL.

Pre-emptive treatment after the detection of minimal residual disease post-transplantation should be considered, although methods of monitoring for minimal residual disease of ATL have not been well established after allo-HSCT. In patients in whom HTLV-1 proviral load (PVL) and/or sIL-2R levels begin to increase (no clear threshold exists to detect patients who are at high risk of relapse) chimerism analysis should be considered as well as early taper of immunosuppression and donor lymphocyte infusions.

Kinetic changes in PVL post–allo-HSCT are variable, but three patterns have been observed in patients with full donor chimerism. The first pattern, observed in recipients of allo-HSCT from infected or uninfected donors, was that changes became undetectable after allo-HSCT and remain so. The second pattern, from uninfected donors, was that PVL became undetectable but returned to detectable levels thereafter, usually 6 to 12 months post–allo-HSCT. The third pattern was in those who had detectable PVL throughout.^59^ Strategies have been undertaken in the United Kingdom and France to minimize neoinfection of donor stem cells after allo-HSCT, including the addition of integrase inhibitors, such as raltegravir at engraftment, or close PVL monitoring and the early addition of zidovudine, although there is no published evidence to support this approach.

### Consensus Statements

1. Up-front allo-HSCT should be considered for all suitable patients with aggressive ATL.
2. Early referral to a transplantation center is recommended.
3. HTLV-1 seronegative donors are preferred to reduce the risk of donor-derived ATL.
4. Mogamulizumab should not be used within 50 days of allo-HSCT.

## ELDERLY AND NON–TRANSPLANTATION-SUITABLE PATIENTS WITH AGGRESSIVE ATL

A retrospective study of elderly patients with aggressive ATL (age > 70 years) observed that elderly patients who were treated with the VCAP-AMP-VECP regimen as initial treatment demonstrated OS that was similar to that in the trial reports of patients age 56 to 70 years.^23^ The regimen was modified with dose reductions that were typically between 50% and 80% in 31 (91%) of 34 patients , and overall response rate [complete response (CR) or partial response (PR)] was 75% after two or three cycles of VCAP-AMP-VECP treatment; however, the completion rate of planned chemotherapy was only 19%. Eleven (32%) of 34 patients who achieved objective responses to initial treatment were switched to an oral maintenance chemotherapy regimen that contained oral etoposide or sobuzoxane and/or prednisolone therapy. With the exception of platelet count, there were no significant differences in the background between patients who were treated with the maintenance treatment and those not. Median survival time and 2-year OS rate of those who received maintenance oral chemotherapy were 16.7 months and 33%, respectively, which suggests that such a treatment strategy is a viable option for elderly and/or non–transplantation-suitable patients, warranting prospective trials.^23^

Alternatively, dose-reduced CHOP-14–like regimens could be considered in elderly and/or non–transplantation-suitable patients, as the subgroup analysis of the JCOG9801 trial demonstrated that the OS rate was similar between VCAP-AMP-VECP and CHOP-14 regimens in patients age 56 to 70 years in contrast to the superiority of the VCAP-AMP-VECP regimen in patients younger than 56 years of age with a 45% OS rate at 1 year.^12^

Combination mogamulizumab and chemotherapy (eg, dose-reduced VCAP-AMP-VECP regimen or CHOP like) could be considered as initial therapy for such patients, although the combination was only evaluated in relatively nonelderly patients.^21^ Despite the lack of published reports on AZT/IFN outcomes for elderly patients, as with younger patients, this combination should be considered as a first-line option followed by maintenance where available.^14,22^

Given the lack of a preferred or compelling treatment strategy for elderly patients with aggressive ATL, alternative options, such as the selection of less intensive regimens that can be used in a continuous fashion, maintenance strategies using oral agents (single-agent etoposide or cyclophosphamide, etoposide, prednisolone [CEP]), or AZT/IFN, may be appropriate. There may be future roles for monoclonal antibodies (eg, anti-CCR4, anti-CD30, or small molecules, such as lenalidomide) as a maintenance strategy, but these require additional evaluation before recommendations can be made.

### Consensus Statement

1. Less intensive induction therapy with or without maintenance therapy with either oral chemotherapy or AZT/IFN may be appropriate for elderly and/or non–transplantation-suitable patients with aggressive ATL.

## STRATEGY OF TARGETING THERAPY: PRECLINICAL DATA

Several antiretrovirals used for HIV have demonstrated proven efficacy against HTLV-1 in tissue culture, including the reverse transcription inhibitors zidovudine and tenofovir, as well as the integrase inhibitor raltegravir.^62,63^ However, whether these agents work in vivo via an antiviral effect has remained controversial. Several studies have shown little, if any, viral structural, enzymatic, or regulatory gene expression in ATL, with the exception of the antisense gene *HBZ*,^5,64,65^ and it remains controversial whether the continued expression of non-*HBZ* viral genes is required for tumor maintenance. However, recent data demonstrate that the extinction of Tax expression leads to total growth inhibition of ATL-derived cells even if Tax protein is undetectable at baseline.^66,67^ A possible explanation for these contradictory results is the recent demonstration on the basis of single-cell analysis of transient bursts of Tax oncoprotein, and presumably other viral proteins, which has led to the suggestion that transient Tax expression is essential for ATL survival.^66,67^ Thus, immunotherapy against Tax might be theoretically promising. A recent phase I study that used vaccination with Tax peptide-pulsed dendritic cells in three patients with aggressive ATL in PR or stable disease after chemotherapy demonstrated an induction of immune response against Tax with promisingly long-term remission.^68^ This warrants larger studies to demonstrate clinical effectiveness.

Although the combination of AZT/IFN has shown activity, it is uncertain whether these drugs function via antiviral activity in cells in the tumor or microenvironment or through mechanisms other than antiviral activity.^69^ Of note, doses of AZT used for the treatment of ATL—600 to 900 mg/d and up to 3,000 mg/d in certain reports—are generally higher than those used for HIV treatment (600 mg/d) although the inhibitory concentrations of AZT for HTLV and HIV replication are similar.^70-72^ It is also unclear whether reactivation of HTLV-1 expression in malignant or nonmalignant cells after induction therapy leads to chemotherapy resistance^73^; however, it is notable that a recent chemotherapy trial that included raltegravir demonstrated results that were similar to a trial that lacked antiviral agents during the same induction therapy.^37^ Conversely, a recent report demonstrated that the combination AZT/IFN induced a significant inhibition of HTLV-I reverse transcription activity in responding ATL patients but not in resistant patients.^74^ These results are in line with a direct antiviral effect, likely in the HTLV-1–infected nonmalignant cells, which may play a major role in the survival of ATL cells.

New insights into the molecular biology of ATL have provided ideas for future clinical trials. Although there was no detectable viral Tax expression in ATL samples, many of the cellular proteins affected by Tax (Tax interactome) were found to be mutated in ATL.^5^ These include genes that are involved in T-cell receptor pathway activation, including phospholipase γ, protein kinase Cβ, caspase recruitment domain-containing protein 11, and interferon regulatory factor 4, affecting the nuclear factor κB (NF-κB) pathway, which is activated in ATL and contributes to cell proliferation and resistance to apoptosis. Inhibitors of these pathway mediators, including protein kinase C or NF-κB inhibitors, could be useful in ATL therapy; however, a trial that included bortezomib, as an NF-κB inhibitor with induction chemotherapy, did not provide significant benefit.^37^ IRF4 expression could have oncogenic potential in ATL and may be associated with interferon resistance,^75^ which warrants therapeutic targeting of its function. Of note, lenalidomide, an active agent in ATL, may function via the inactivation of IRF4 by enhancing the degradation of the IRF4 gene activators Ikaros and Aiolos.^76^

Genomic studies of ATL have revealed high rates of genetic damage compared with other hematopoietic malignancies.^5^ In addition, genes that are involved in antigen presentation and immune surveillance are often mutated,^5^ including aberrant programmed death ligand-1 expression.^77^ These findings suggest that immune checkpoint therapy could play a role in ATL treatment. Currently, a trial of a programmed death-1 inhibitor, nivolumab, is underway in Japan (Data Supplement), although a study in the United States was recently halted as a result of concerns over accelerated disease progression.^38^

## NEW AGENTS FOR ATL

Recently, two new agents, mogamulizumab, an anti-CCR4 monoclonal antibody, and lenalidomide, an immunomodulatory drug, have been approved for the treatment of ATL in Japan after pivotal trials for relapsed aggressive ATL^27,28^ Mogamulizumab has been also approved for the treatment of newly diagnosed aggressive ATL in combination with intensive chemotherapy.^21^

CCR4 is expressed in neoplastic cells in approximately 90% of ATL cases and mutated in 26%. This expression has been associated with cutaneous manifestations and poor prognosis.^78^ In phase I and II studies conducted in Japan, a response rate of approximately 50% was observed with manageable toxicities, including moderate-to-severe skin reactions and other immunopathology, even in nontransplantation patients, possibly by depleting nontumor regulatory T cells.^24,28^ CR rates varied among target lesions—rates were high in peripheral blood, intermediate in skin, and low in lymph nodes. Median PFS and OS were 5.2 months and 13.7 months, respectively. The findings of a subsequent randomized phase II study of intensive chemotherapy (modified LSG15) with or without mogamulizumab for the treatment of untreated aggressive ATL has recently been reported.^21^ The combination was well tolerated and produced a higher CR rate compared with chemotherapy alone (52% [95% CI, 33% to 71%] *v* 33% [95% CI, 16% to 55%]). However, PFS and OS were identical in both arms, although the sample size and duration of follow-up were relatively short and median OS was not reached in either arm. Recently, a phase II trial in Europe, the United States, and South America of single-agent mogamulizumab (1 mg/kg per week for 4 weeks, followed by 1 mg/kg every 2 weeks until progressive disease) versus investigator’s choice of salvage chemotherapy for relapsed aggressive ATL revealed a 15% confirmed overall response rate in the mogamulizumab arm versus 0% for investigator’s choice.^25^

A phase I study of lenalidomide demonstrated preliminary antitumor activity in patients with relapsed ATL or PTCL at a higher dose than that used for the treatment of multiple myeloma.^26^ A subsequent phase II study evaluated the efficacy and safety of lenalidomide monotherapy at an oral dose of 25 mg/d continuously until progressive disease or unacceptable toxicity in patients with relapsed or recurrent aggressive ATL.^78^ Objective responses were noted in 11 of 26 patients [objective response rate, 42% (95% CI, 23% to 63%)], including four patients with CR and one with unconfirmed CR. Rate of disease control, including overall response and stable disease, was 73%. Median time to response and duration of response were 1.9 months and not estimable, respectively. Median PFS and OS were 3.8 months and 20.3 months, respectively. The most frequent grade 3 or greater adverse events were neutropenia (65%), leukopenia (38%), lymphopenia (38%), and thrombocytopenia (23%), which were all manageable and reversible.^26^ In conclusion, lenalidomide demonstrated clinically meaningful antitumor activity and an acceptable toxicity profile in patients with relapsed or recurrent aggressive ATL. Lenalidomide also demonstrated activity with CNS involvement by diffuse large B-cell lymphoma,^79^ which could have implications for its use in ATL despite the lack of data in the previous trial as CNS involvement was an exclusion criterion.

Other US Food and Drug Administration (FDA)–approved drugs are now available for the treatment of relapsed/refractory PTCL and have been used for ATL in the United States, including the antifolate agent pralatrexate and the histone deacetylase inhibitors belinostat and romidepsin (listed alphabetically). Mogamulizumab has recently received FDA approval in MF or Sézary syndrome after at least one prior therapy (August 2018). Although not FDA approved, current NCCN guidelines support the use of brentuximab vedotin in CD30^+^ cases (NCCN grade 2A). Pralatrexate was included as an investigator choice drug in the recently completed randomized phase II trial of single-agent mogamulizumab versus salvage chemotherapy for the treatment of relapsed aggressive ATL, but no confirmed responses were reported.^25^ Histone deacetylase inhibitors are known to activate HTLV-1 expression; therefore, these drugs might be used with caution in ATL, with consideration given to the addition of an antiretroviral agent (ie, AZT) and under clinical trials desirably.^80^ Epstein-Barr virus reactivation in patients with extranodal natural killer/T-cell lymphoma was reported as a previously unrecognized serious adverse event in a pilot study with romidepsin.^81^

### Consensus Statement

1. All patients should be considered for entry into clinical trials where available.

## PERSPECTIVES

Whereas the backbone of ATL treatment has remained largely unchanged since the 2009 consensus report, there have been several advances in the clinical management of these patients, particularly for patients treated in Japan. These include the increased role of allo-HSCT after first-line treatment and the use of mogamulizumab and lenalidomide as novel single agents, both licensed in Japan. The precise roles of these agents and others, such as brentuximab vedotin for CD30^+^ ATL, remains incompletely understood. Clinical trials are required to understand the roles of these agents in the front-line setting, including indolent subtypes, in maintenance therapy for transplantation-ineligible patients, in maintenance therapy after allo-HSCT, and in the prevention or treatment of CNS disease. As ATL is characterized by early relapses, clinical trials of therapies that might eradicate minimal residual disease are warranted. Preliminary data from France have shown that combining arsenic trioxide with low-dose interferon is feasible and an effective consolidation therapy that is capable of selectively eliminating the malignant clone and restoring oligoclonal architecture. This has raised hopes that the extinction of viral replication (AZT) and Tax degradation (arsenic/IFN) may eradicate the disease.^15,16,82,83^ Addition of arsenic trioxide to IFN/AZT was promising in a phase II study for the treatment of chronic ATL.^16^

Since 2009, there has been more widespread use of PET/CT in the diagnosis and follow-up of patients with non-Hodgkin lymphoma. ATL is frequently associated with extranodal disease, and it might be recommended that PET/CT be used at diagnosis, where available, and that PET/CT assessment should be incorporated in future studies. The significance of negative interim PET/CT should be evaluated as it is not yet clear how this might affect treatment strategy.

Reported prognostic factors at diagnosis (eg, IRF4 expression, TP53 mutation/deletion, and other genetic markers) require validation in the context of treatment with AZT/IFN, chemotherapy, or active monitoring to assist in tailored treatment decisions. New methodologies to detect clonal HTLV minimal residual disease have been established using next-generation high-throughput sequencing methods, and the challenge now remains to validate these observations within clinical trials and bring them into the clinical domain in a rapid, cost-effective manner.

ATL continues to have a dismal prognosis with current therapies, and clinical trials that incorporate molecular and prognostic factors will remain paramount to advances in ATL treatment. We have proposed a strategy for ATL treatment stratified by subtype classification, including an updated opinion on the management of patients with tumorous skin lesions. Future clinical trials should remain a priority to ensure that the consensus is continually updated to establish evidence-based practice guidelines.
